# Mechanisims of asthma and allergic disease – 1093. A novel human anti-VCAM-1 Monoclonal antibody Ameliorates airway inflammation and remodeling in murine asthma model

**DOI:** 10.1186/1939-4551-6-S1-P89

**Published:** 2013-04-23

**Authors:** Jae-Hyun Lee, Jung-Ho Sohn, Su Yeon Ryu, Kyung D Moon, Chein-Soo Hong, Jung-Won Park

**Affiliations:** 1 Div. of Allergy and Immunology, Dept. of Internal Medicine, Yonsei University College of Medicine, Seoul, South Korea; 2 Department of Life Science, Research Institute for Natural Sciences, Hanyang University, Seoul, South Korea; 3 Hanwha Chemical R&D Center, Daejeon, South Korea

## Background

Asthma is a chronic inflammatory disease induced by Type 2helper T cells (Th2) and eosinophils. Vascular cell adhesion molecule-1(VCAM-1) is the regulatory receptor implicated with recruiting eosinophils andlymphocytes to pathologic site in asthma. A monoclonal antibody (mAb)against VCAM-1 may attenuate allergic inflammation and pathophysiologicfeatures of asthma. Weevaluated whether a recently developed human anti-VCAM-1mAb can inhibit pathophysiologic features of asthma in a murine asthma modelinduced by ovalbumin (OVA).

## Methods

We evaluated whether human anti-VCAM-1 mAb binds to human ormouse VCAM-1. Leukocyte adhesion inhibition assay was performed toevaluate the *in vitro* blocking activity of human anti-VCAM-1 mAb. OVAsensitized BALB/c mice were treated with human anti-VCAM-1 mAb orisotype control Ab before intranasal OVA challenge. We evaluated airwayhyperresponsiveness (AHR) and cell counts in bronchoalveolar lavage (BAL)fluid, measured inflammatory cytokines, and examined histopathologicalfeatures, including VCAM-1 immunohistochemistry.

## Results

The human anti-VCAM-1 mAb bound to human and mouse VCAM-1molecules and inhibited adhesion of human leukocytes *in vitro*. AHR andinflammatory cell counts in BAL fluid were reduced in mice treated withhuman anti-VCAM-1 mAb as compared to a control Ab. The levels ofinterleukin (IL)-5 and IL-13, and transforming growth factor-β in lung tissuewere decreased in treated mice. Human anti-VCAM-1 mAb reduced goblet cellhyperplasia and peribronchial fibrosis. *In vivo* VCAM-1 expression decreasedin treated group.

## Results

Human anti-VCAM-1 mAb can attenuate allergic inflammationand pathophysiological features of asthma in OVA induced murine asthmamodel. This data suggested that human anti-VCAM-1 mAb could be an additionalanti-asthma therapeutic medicine.

